# Polysaccharide compositions of collenchyma cell walls from celery (*Apium graveolens* L.) petioles

**DOI:** 10.1186/s12870-017-1046-y

**Published:** 2017-06-15

**Authors:** Da Chen, Philip J. Harris, Ian M. Sims, Zoran Zujovic, Laurence D. Melton

**Affiliations:** 1 School of Chemical Sciences, University of Auckland, Auckland, New Zealand; 2 School of Biological Sciences, University of Auckland, Auckland, New Zealand; 3 Ferrier Research Institute, Victoria University of Wellington, Wellington, New Zealand; 4 NMR Centre, School of Chemical Sciences, University of Auckland, Auckland, New Zealand

**Keywords:** Collenchyma, Anatomy, Plant cell walls, Pectin, Hemicelluloses, Cellulose, Polysaccharide linkage analysis, Polysaccharide mobility, Solid-state ^13^C NMR, Variable pressure scanning electron microscopy

## Abstract

**Background:**

Collenchyma serves as a mechanical support tissue for many herbaceous plants. Previous work based on solid-state NMR and immunomicroscopy suggested collenchyma cell walls (CWs) may have similar polysaccharide compositions to those commonly found in eudicotyledon parenchyma walls, but no detailed chemical analysis was available. In this study, compositions and structures of cell wall polysaccharides of peripheral collenchyma from celery petioles were investigated.

**Results:**

This is the first detailed investigation of the cell wall composition of collenchyma from any plant. Celery petioles were found to elongate throughout their length during early growth, but as they matured elongation was increasingly confined to the upper region, until elongation ceased. Mature, fully elongated, petioles were divided into three equal segments, upper, middle and lower, and peripheral collenchyma strands isolated from each. Cell walls (CWs) were prepared from the strands, which also yielded a HEPES buffer soluble fraction. The CWs were sequentially extracted with CDTA, Na_2_CO_3_, 1 M KOH and 4 M KOH. Monosaccharide compositions of the CWs showed that pectin was the most abundant polysaccharide [with homogalacturonan (HG) more abundant than rhamnogalacturonan I (RG-I) and rhamnogalacturonan II (RG-II)], followed by cellulose, and other polysaccharides, mainly xyloglucans, with smaller amounts of heteroxylans and heteromannans. CWs from different segments had similar compositions, but those from the upper segments had slightly more pectin than those from the lower two segments. Further, the pectin in the CWs of the upper segment had a higher degree of methyl esterification than the other segments. In addition to the anticipated water-soluble pectins, the HEPES-soluble fractions surprisingly contained large amounts of heteroxylans. The CDTA and Na_2_CO_3_ fractions were rich in HG and RG-I, the 1 M KOH fraction had abundant heteroxylans, the 4 M KOH fraction was rich in xyloglucan and heteromannans, and cellulose was predominant in the final residue. The structures of the xyloglucans, heteroxylans and heteromannans were deduced from the linkage analysis and were similar to those present in most eudicotyledon parenchyma CWs. Cross polarization with magic angle spinning (CP/MAS) NMR spectroscopy showed no apparent difference in the rigid and semi-rigid polysaccharides in the CWs of the three segments. Single-pulse excitation with magic-angle spinning (SPE/MAS) NMR spectroscopy, which detects highly mobile polysaccharides, showed the presence of arabinan, the detailed structure of which varied among the cell walls from the three segments.

**Conclusions:**

Celery collenchyma CWs have similar polysaccharide compositions to most eudicotyledon parenchyma CWs. However, celery collenchyma CWs have much higher XG content than celery parenchyma CWs. The degree of methyl esterification of pectin and the structures of the arabinan side chains of RG-I show some variation in the collenchyma CWs from the different segments. Unexpectedly, the HEPES-soluble fraction contained a large amount of heteroxylans.

**Electronic supplementary material:**

The online version of this article (doi:10.1186/s12870-017-1046-y) contains supplementary material, which is available to authorized users.

## Background

Collenchyma is a type of supporting tissue commonly found in stems, petioles, leaves and floral segments of many herbaceous and woody plants, particularly in eudicotyledons [1, 2]. This tissue is commonly located peripherally in organs, where it may occur either immediately below the epidermis, or separated by several layers of parenchyma cells (Additional file 1: Figure S1). In addition to its peripheral location, collenchyma is often associated with vascular bundles [1–3]. A predominant feature of collenchyma cells is their unevenly thickened walls that are usually regarded as primary [2]. Based on the positions of the wall thickenings, collenchyma can be divided into four main types: angular collenchyma, which is the commonest type and has thickening mainly in the cell corners, e. g. in the petioles of celery (*Apium graveolens*), stems of potato (*Solanum tuberosum*) and deadly nightshade (*Atropa belladonna*) [4]; tangential collenchyma has the inner and outer tangential walls thickened more heavily than the radial ones, e.g. in stems of elder (*Sambucus nigra*) [5]; annular collenchyma has the walls uniformly thickened, e.g. in the petioles of cow parsnip (*Heracleum lanatum*) and petioles of sweetgum (*Liquidamber styraciflua*) [6]; lacunar collenchyma has intercellular spaces adjacent to the thickened walls, e.g. in the petioles of winter heliotrope (*Petasites fragrans*) [6].

Plant cell walls are composed of cellulose microfibrils embedded in a matrix composed mostly of polysaccharides, but also proteins and glycoproteins, together with phenolic and inorganic compounds [7]. The composition of the matrix varies depending on the cell type, stage of development and plant taxon [7, 8]. Much of what is known about the compositions of mature primary cell walls of eudicotyledons comes frequently from cell-wall preparations derived mostly from parenchyma cells. Analyses of such cell-wall preparations have shown that the matrix polysaccharides are composed predominantly of pectic polysaccharides (pectins) [7, 8]. Pectic polysaccharides consist of domains, of which the most common domain is homogalacturonan (HG) comprising (1 → 4)-linked α-D-galacturonosyl residues, which may be methyl or acetyl esterified to varying extents. The next most abundant domain is usually rhamnogalacturonan I (RG-I), which consists of alternating (1 → 4)-linked α-D-galacturonosyl and (1 → 2)-linked α-L-rhamnosyl residues as a backbone; up to 80% of the Rha*p* residues may have side chains consisting of (1 → 5)-α-L-arabinans, (1 → 4)-β-D-galactans and arabino-4-galactans; the α-D-galacturonosyl residues may be acetylated [9]. Smaller proportions of a third domain, rhamnogalacturonan II (RG-II), consistently occur in pectic polysaccharides. Yet another domain, xylogalacturonan (XGA), occurs in some pectic polysaccharides.

In addition to the pectic polysaccharides, the matrix polysaccharides include xyloglucans, which in most eudicotyledons are fucogalactoxyloglucans [10–13]. These have a linear backbone of (1 → 4)-linked β-D-Glc*p* residues, three quarters of which bear side chains of single α-D-Xyl*p* residues, β-D-Gal*p*-(1 → 2)-α-D-Xyl*p*- or α-L-Fuc*p*-(1 → 2)-β-D-Gal*p*-(1 → 2)-α-D-Xyl*p*. The detailed arrangements of the side chains have been described [10–13]. The matrix also contains small amounts of heteroxylans (HXs) and heteromannans (HMs) [7, 8, 11, 14].

Although the structure of the cellulose microfibrils of collenchyma cell walls has been studied extensively [15–17] little is known in detail about the matrix polysaccharides. Some information has been obtained using solid-state NMR spectroscopy and this suggested a matrix composition similar to that found in parenchyma walls [18, 19]. These NMR studies were all done on collenchyma cell walls from celery petioles. In addition, information about the occurrence of matrix polysaccharides in collenchyma walls has been obtained by immunofluorescence labelling of transverse sections of tomato (*Solanum lycopersicum*) petioles [20], tobacco (*Nicotiana tabacum*) stems [21–23] and elder (*Sambucus nigra*) stems [2]. These showed the presence of the following polysaccharides: HG and RG-I side chains [(1 → 5)-α-L-arabinans and (1 → 4)-β-D-galactans], xyloglucans, heteroxylans and heteromannans.

In the present study, we aimed to determine the polysaccharide compositions, possible structures and relative mobilities of polysaccharides in collenchyma cell walls obtained from peripheral collenchyma strands of fully elongated celery petioles, but not those strands associated with the vascular bundles. As part of this study, we investigated the growth of celery petioles. We also examined transverse sections of the collenchyma strands using bright-field light microscopy and variable pressure scanning electron microscopy (VPSEM). For the investigation of the polysaccharide compositions, we isolated collenchyma cell walls, which also resulted in a HEPES soluble fraction. The CWs were sequentially fractionated. The monosaccharide and glycosyl linkage compositions of the polysaccharides were determined, both for the whole cell walls and the fractions. The mobilities of the polysaccharides in the cell walls were studied by cross-polarisation/magic angle spinning (CP/MAS) and single pulse excitation/magic angle spinning (SPE/MAS)^13^C nuclear magnetic resonance (NMR) spectroscopy.

## Methods

### Plant material

Celery (*Apium graveolens* L*.*, ‘Tango’) was grown from seed in an unheated glasshouse at Clark Nurseries Ltd., Pukekohe, New Zealand (geographical location 37° 20′ S, 174° 88′ E), and was harvested on 21st August 2015, after 6-months growth. Celery without evident damage or malnutrition was chosen for experiments. Mature, fully elongated petioles (35–40 cm long) from the outer region of the leaf bundles were cut 3–5 cm from the base and at the top immediately below the junction of the first leaflet. The excised petioles were then cut into three segments of equal lengths, referred to as the upper (adjacent to the junction with the first leaflet), middle and lower segments. The epidermis of each segment, cooled on ice to reduce enzyme activities, was carefully removed and the peripheral (subepidermal) collenchyma strands detached using forceps, and stored at −80 °C.

**Fig. 1:**
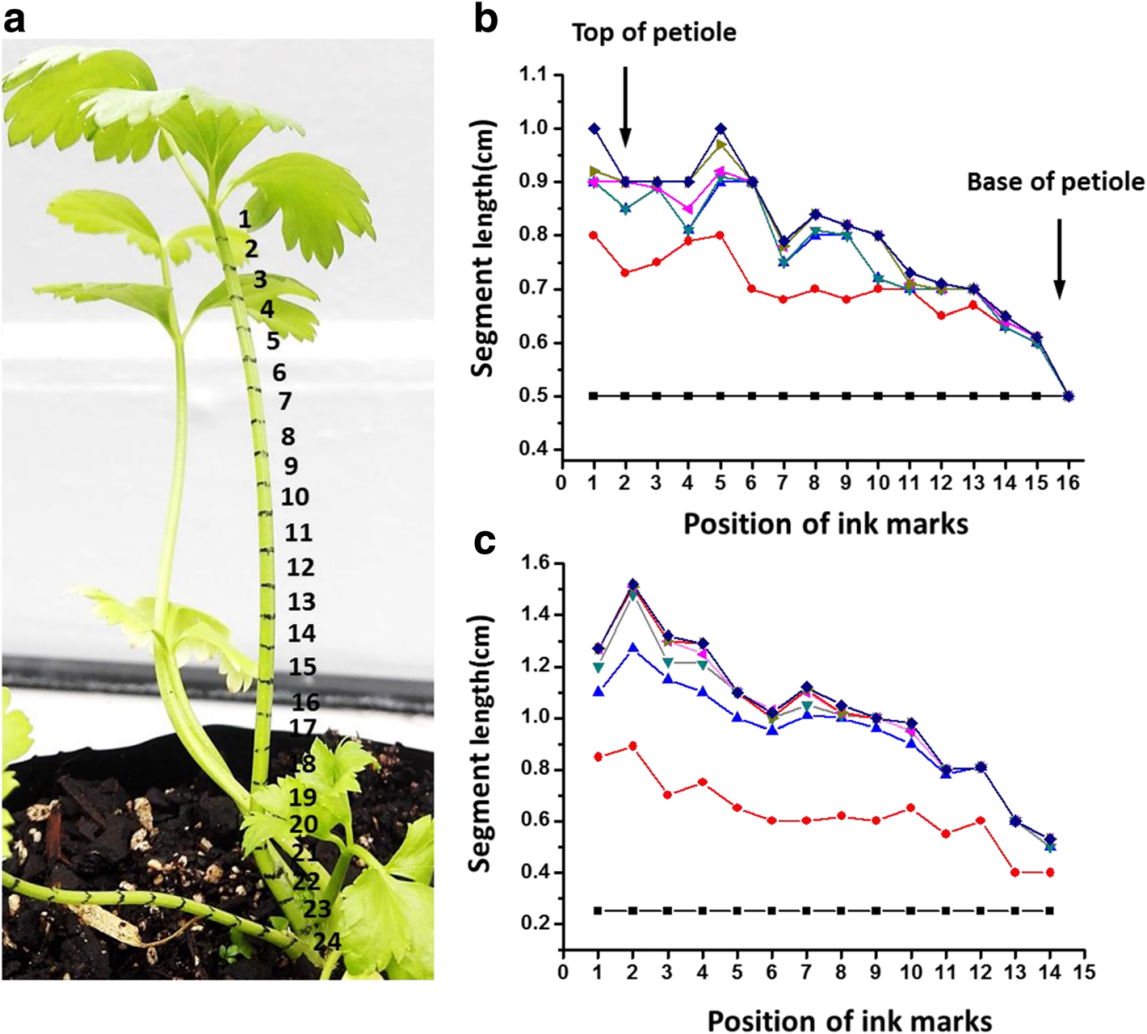
The position of ink marks (**a**) and representative growth of short (**b**) and long celery petioles (**c**). **a** Shows a celery leaf with a long petiole marked at 0.5 cm interval with a *black marker*. The mark position (*right side number*) was set from upper to lower segments of celery petiole. **b** and **c** show the interval lengths from the long (7.5 cm) and short (3.3 cm) petioles after Day 1 (marking day) ; Day 5 ; Day 13 ; Day 21 ; Day 28 ; Day 36 ; Day 50 . These are representative petioles; a total of 13 short and 13 long petioles were studied

For the growth experiment, thirteen immature celery plants (5–10 cm high), provided by Clark Nurseries Ltd., were transferred to a heated (minimum 18 °C and maximum 35 °C) glasshouse at The School of Biological Sciences, University of Auckland, provided with 16 h light, watered daily and fertilized weekly with Nitrosol® (Rural Research Ltd., Auckland, New Zealand). A waterproof, black marker pen was used to mark at 0.5 and 0.25 cm intervals along the outer bundle petioles (7–15 cm long) and inner petioles (2–6 cm long), respectively; (Fig. 1a) 26 petioles were marked in total. The day of the marking was defined as day 1, and changes in the distances between the marks were recorded on day 6, 13, 21, 28, 36, 50 or 6, 13, 21, 28, 42, depending on the growth rate of the petioles.

### Bright-field light microscopy and variable pressure scanning electron microscopy (VPSEM)

Transverse sections were cut with a razor blade from midway along each segment, stained with a 0.1% (*w*/*v*) aqueous solution of toluidine blue O for 1 min, washed with water, and examined by bright-field light microscopy (Model DMR500, Leica, Wetzlar, Germany). Five peripheral collenchyma strands were randomly chosen from three different petioles from different plants. The cross sectional area and number of cells in each strand were determined using Image J software (NIH, New York, USA). The average cross sectional cell area was calculated by dividing the cross sectional area of each strand by the number of cells in the strand.

For VPSEM, cubes of tissue (~10 mm^3^) were cut midway along each segment, rinsed with 20 mM potassium HEPES buffer (pH 6.7) containing 10 mM DTT and then water. The tissue cubes were examined by VPSEM using a FEI Quanta 200F ESEM (Hillsboro, Oregon, USA). The temperature was kept at 2 °C, the initial chamber pressure was 5.3 Torr, and the accelerating voltage was 20 KV.

### CW isolation and fractionation

Cell walls were isolated as described by Melton & Smith [24] with minor modifications. Collenchyma strands were detached from petioles as described above, and stored at −80 °C until used. Frozen collenchyma strands were ground to a powder under liquid nitrogen using a mortar and pestle, then homogenized using a blender (Ultra Turrax, Staufen, Germany) in cold 20 mM potassium HEPES buffer (pH 6.7) containing 10 mM DTT, followed by further grinding (3 × 2 min) in a ring grinder (Rocklabs, Auckland, New Zealand). Cell breakage was checked by bright-field light microscopy after staining with an aqueous solution of 0.2% (*w*/*v*) Ponceau 2R containing 2 drops of 18 M sulfuric acid per 100 mL solution [25]. The slurry was filtered with a nylon mesh (11 μm pore size) and washed four times with the buffer. The filtrate (HEPES fraction) and the residue (CWs) on the mesh were collected and dialyzed against water (molecular weight cut-off 12–14 kDa) for 5 days at 4 °C with three changes of water per day. The HEPES fraction and CWs were freeze dried. The freeze dried CWs were sequentially extracted twice each with 50 mM CDTA in 50 mM, potassium acetate buffer (pH 6.5) for 6 h at room temperature (CDTA fraction), 50 mM Na_2_CO_3_ containing 20 mM NaBH_4_ for 16 h at 4 °C, then 4 h at room temperature (Na_2_CO_3_ fraction), 1 M KOH containing 20 mM NaBH_4_ for 4 h at room temperature (1 M KOH fraction), 4 M KOH containing 20 mM NaBH_4_ 4 h at room temperature (4 M KOH fraction). The CDTA fractions were dialyzed against 0.1 M ammonium acetate for 3 days with three changes of buffer per day then water. All the other fractions and residues (Residue fraction) were dialyzed (molecular weight cut-off 12–14 kDa) against water for 5 days at 4 °C, and the retentates concentrated and freeze-dried. The experiments were done in duplicate.

### Monosaccharide compositions

CWs and fractions (~2 mg) were hydrolyzed with 2 M trifluoroacetic acid (TFA) (2 mL) at 120 °C for 1 h [26] to hydrolyze the non-cellulosic polysaccharides [27]. Hydrolysis was also carried out using a two-stage H_2_SO_4_ procedure [28, 29]. The CWs and fractions (~2 mg) were mixed with H_2_SO_4_ (0.25 mL, 72% *w*/*w*) at 30 °C for 3 h, water (2.75 mL) was then added and heated at 100 °C for 3 h to hydrolyze cellulose and non-cellulosic polysaccharides. After cooling, the hydrolysate was neutralized with 15 M ammonia (0.6 mL), *myo*-inositol (20 μL of a 2 mg/mL solution) added, filtered through a 0.22 μm PTFE filter, and dried in a stream of N_2_ at 50 °C. The same procedure was also conducted on the TFA hydrolysate, but without the addition of ammonia. The monosaccharides in the dried hydrolysates were reduced with NaBH_4_ (10 mg/mL) in 2.5 M ammonium hydroxide (2.5 mL) for 1.5 h at room temperature, then neutralized with 17.4 M acetic acid (50 μL). To remove borate, acidic methanol (0.5 mL, methanol: acetic acid 9:1, *v*/*v*) was added and evaporated to dryness in a stream of N_2_ at 50 °C. This was repeated a further three times. Anhydrous methanol (0.5 mL) was added and evaporated to dryness under the same conditions three times. The alditols were then acetylated using acetic anhydride (2 mL) with 1-methylimidazole (0.2 mL) as the catalyst for 10 min at room temperature. Water (5 mL) was then added to destroy excess acetic anhydride. After cooling, the alditol acetates were extracted twice with dichloromethane (DCM) (1 mL). The combined DCM extracts were washed with water (4 mL) five times before being evaporated to dryness in a stream of N_2_. DCM (1 mL) was added and 0.5 μL injected onto a Hewlett Packard Model HP 6890 gas chromatography system (Palo Alto, California, USA) equipped with a fused silica capillary column BPX-70 (25 m long, 0.33 i.d. and 0.25 μm film thickness) (SGE Analytical Science, Melbourne, Australia) and a flame ionization detector [30]. The oven temperature was set at 38 °C for 1 min, then increased to 170 °C at 50 °C/min and further increased to 230 °C at 2 °C/min and held for 5 mins. Helium was used as the carrier gas. Total uronic acids were measured by the *m*-hydroxydiphenyl method with D-galacturonic acid as the standard [31]. All determinations were done in triplicate.

### Glycosyl linkage analysis

Prior to glycosyl linkage analysis, uronic acid residues were reduced to their dideuterio-labelled neutral sugars as described by Sims and Bacic [32] with slight modifications. Cell walls and fractions (~5 mg) were dispersed in 1 mL 50 mM MES acid (1 mL, pH 4.75) buffer, uronic acids were activated by addition of 1-cyclohexyl-3-(2-morpholinoethyl)-carbodiimide-*metho*-*p*-toluenesulfonate (400 μL, 500 mg/mL) and reduced overnight with NaBD_4_. The samples were dialysed (molecular weight cut-off 6–8 kDa) against water, freeze-dried and then reduced a second time as above.

The carboxyl-reduced samples (~0.5 mg, in duplicate) were dispersed in DMSO (200 μL) by stirring for 24 h under argon to aid swelling of the insoluble cell wall and residue samples, and methylated using the method of Ciucanu and Kerek [33]. After extraction into chloroform, the methylated samples were hydrolysed with 2.5 M TFA (2.5 M, 500 μL, 120 °C, 1 h), neutralised with 1 M NH_4_OH (100 μL) and reduced by 1 M NaBH_4_ in 1 M NH_4_OH (100 μL) overnight at 25 °C. The reaction was stopped by adding glacial acetic acid (50 μL), washed with acidic methanol and evaporated under a stream of air at 40 °C. The resulting alditols were acetylated and extracted into DCM. The DCM was washed twice with water and evaporated under N_2_. Subsequently, the dried partially methylated alditol acetates (PMAAs) were dissolved in acetone and 0.2 μL was injected onto a GC fitted with a BPX90 fused silica capillary column (SGE Analytical Science, Melbourne, Australia; 30 m × 0.25 mm i.d., 0.25 μm film thickness) with the temperature programme of 80 °C (held for 1 min) to 130 °C at a rate of 50 °C min^−1^, then to 230 °C at a rate of 3 °C min^−1^ and detected by MS using a Hewlett Packard 5973 MSD. In some cases, where PMAAs were observed to co-elute on the BPX90 column, additional analyses were completed on an Agilent HP-5MS column (30 m × 0.25 mm i.d., 0.25 μm film thickness; Agilent, Santa Clara, CA, USA). Identifications were based on peak retention times relative to an internal standard, *myo*-inositol, and on comparisons of electron impact spectra with the spectra obtained from reference PMAA standards prepared by the method of Doares et al. [34].

### Degrees of methyl and acetyl esterification

The degree of esterification was determined by GC with a flame ionization detector as described by Nunes et al. [35] with minor modifications. Briefly, CWs or fractions (~ 5 mg) were weighed into Eppendorf tubes (1.5 mL), water (0.6 mL), 5 mg/mL 1-propanol water mixture (30 μL) and 2 M NaOH (0.2 mL) were added. The mixture was incubated (2 h at 25 °C), neutralized with 2 M HCl, centrifuged (25,000×g, for 10 mins), filtered (PTFE filter 0.22 μm pore size) and the filtrate (1 μL) injected onto a Agilent 6890 gas chromatograph equipped with a DB-WAX column (30 m length, 0.53 mm i.d, 1.0 μm film thickness) (Agilent, Santa Clara, California, USA) and a flame ionization detector. The oven temperature was programmed from 50 °C to 185 °C at 5 °C/min (for 3 min), 20 °C/min (for 4 min), 35 °C/min (for 1 min) and then held for 2.5 min. The flow rate of carrier gas (helium) was 6 mL/min and the detector temperature was 250 °C. Duplicate samples of each fraction or CW were analyzed twice (*n* = 4). Methanol and acetic acid standards was used for calibration. DM = methanol (mol)/UA (mol) in each mg cell wall × 100%; DA = acetic acid (μg)/total sugars in each mg cell wall × 100%.

### Solid-state NMR

CP/MAS and SPE/MAS NMR spectroscopy were carried out as described by Bootten et al. [36]. Freeze dried cell walls were rehydrated to 80% (*v*/v) ethanol, and then air dried to a moisture of 50% (*w*/w) at room temperature. They were then packed in 4-mm diameter zirconia rotors and retained with Kel-F end-caps. The samples were spun at 4 kHz in a Bruker magic-angle spinning double-tuned probe using a Bruker Avance III 500 spectrometer operating at 125.78 MHz for ^13^C frequency. For the RAMP CP–MAS (ramped amplitude cross-polarization magic-angle spinning) approach, the 90° proton preparation pulse was 2.8 μs followed by a 1 ms CP contact time, 26 ms of data acquisition, and a recovery delay of 1 s, before the sequence was repeated. A total of 20,000 transients were used. A SPINAL-64 decoupling scheme was applied during acquisition. SPE/MAS experiments were done with a 2.8 μs ^13^C excitation pulse followed by 26 ms of data acquisition time, and a 0.2 s recovery delay. The recovery delay was chosen to allow observation of signals from the most mobile polysaccharides and to suppress signals from semi-rigid and rigid domains. A total of 335,000 transients were collected. The spectra were referenced to tetramethysilane (TMS) set at 0.00 ppm, and were assigned according to [19, 37, 38].

## Results

### Petiole growth

To determine which regions of celery petioles elongate the most during growth, the intervals between initially equidistant ink marks on long and short petioles from the outside and centre of celery bundles respectively, were measured for up to 50 days. The results showed that when the petioles were young, they elongated throughout their lengths, but later, elongation was increasingly confined to the upper regions, until elongation ceased (Fig. 1b, c).

### Collenchyma anatomy

Bright-field light and VPSE micrographs of transverse sections of subepidermal collenchyma strands midway along the upper, middle and lower petiole segments are shown in Additional file 1: Figure S1. The collenchyma strands in the upper segment had smaller cross-sectional areas with fewer cells than the collenchyma strands in the middle and lower segments (Additional file 2: Figure S2). The collenchyma cells in the upper segment collenchyma strands had thicker walls (Additional files 1 and 2: Figures S1, S2) than those of middle and lower segments.

### Monosaccharide compositions of CWs and fractions

The yields of dry CWs from 100 g of fresh collenchyma strands from upper, middle and lower segments were 12, 11.4 and 9.5 g respectively (Table 1). The HEPES fraction contained the least polysaccharides in all the fractions. The CDTA and Na_2_CO_3_ fractions contributed 27–30% of the total cell-wall polysaccharides. Nearly 10% of the cell-wall polysaccharides were extracted with the 1 M KOH and a larger proportion in the 4 M KOH fraction. Almost 40% of the total dry weight remained in the final residue. Higher yields of cell walls were obtained from collenchyma strands from the upper than from the lower segments, presumably because the cell walls are thicker in the upper segment.

**Table 1:** The yield, monosaccharide compositions (mol%) and degree of esterification of fractions and cell walls from collenchyma strands from different petiole segments, upper (U), middle (M﻿) and lower (L)﻿

Fractions		Yield (%)	Rha	Fuc	Ara	Xyl	Man	Gal	Glc	C-Glc	UA	TS (μg/mg)	DM	DA
HEPES	U	1.6^a^	2.5^a^	1.0^a^	9.7^a^	27.6^a^	2.3^b^	13.1^a^	9.2^a^	-	34.7^a^	338.7^ab^	16.9^a^	6.4^a^
	M	1.7^a^	3.5^b^	0.7^b^	10.8^b^	22.2^b^	1.8^a^	13.7^a^	5.6^b^	-	41.7^b^	346.6^b^	15.6^a^	5.3^a^
	L	1.5^a^	3.6^b^	0.7^b^	11.6^b^	20.1^b^	2.4^b^	15.0^b^	5.9^b^	-	40.7^b^	308.9^a^	19.5^b^	6.0^a^
CW	U	12.0^a^	2.5^a^ (1.6^a^)	0.8^a^ (1.0^a^)	4.4^b^ (4.9^b^)	5.0^a^ (8.0^a^)	1.6^a^ (2.9^a^)	5.1^b^ (5.7^b^)	3.4^a^ (3.2^a^)	32.3^a^ (30.4^a^)	44.9^a^ (42.3^a^)	776.0^a^ (819.7^a^)	25.9^a^	1.6^a^
	M	11.4^ab^	2.0^a^ (1.5^a^)	0.7^a^ (0.9^a^)	3.3^a^ (4.0^a^)	4.1^a^ (7.6^a^)	1.5^a^ (3.0^a^)	3.7^a^ (4.7^ab^)	3.1^a^ (2.9^a^)	37.4^a^ (34.5^c^)	44.2^a^ (40.8^a^)	766.0^a^ (824.9^a^)	16.9^b^	1.4^ab^
	L	9.5^b^	2.1^a^ (1.4^a^)	0.7^a^ (0.9^a^)	3.5^a^ (3.9^a^)	4.5^a^ (7.2^a^)	1.6^a^ (3.0^a^)	3.6^a^ (4.3^a^)	3.3^a^ (3.1^a^)	34.9^a^ (33.0^b^)	45.8^a^ (43.3^a^)	766.6^a^ (807.5^a^)	17.1^b^	1.2^b^
CDTA	U	16.6^a^	1.8^a^	Trace	5.0^a^	1.4^b^	Trace	2.3^a^	1.1^a^	-	88.4^a^	884.3^a^	3.8^a^	0.9^a^
	M	16.9^a^	1.8^a^	Trace	4.3^a^	1.2^a^	0.3	2.0^a^	1.0^a^	-	89.3^a^	963.4^a^	3.2^a^	0.8^a^
	L	20.5^b^	2.0^a^	Trace	4.4^a^	1.2^a^	0.3	2.1^a^	1.0^a^	-	89.1^a^	857.6^a^	5.3^b^	1.0^a^
Na_2_CO_3_	U	12.8^a^	3.7^a^	Trace	7.6^a^	1.9^a^	Trace	4.6^a^	1.0^a^	-	81.1^b^	587.2^a^		
	M	9.6^a^	4.6^b^	Trace	7.5^a^	2.1^a^	Trace	4.5^a^	1.3^a^	-	80.1^b^	509.8^a^		
	L	10.4^a^	5.1^b^	Trace	8.5^a^	2.0^a^	Trace	5.5^a^	2.3^b^	-	76.6^a^	572.0^a^		
1 M KOH	U	13.1^a^	3.1^a^	1.6^b^	5.9^a^	22.3^b^	5.1^a^	8.7^b^	15.5^a^	-	37.8^a^	746.0^a^		
	M	9.0^b^	3.2^a^	1.0^a^	5.3^a^	19.3^a^	5.8^a^	7.0^a^	11.6^a^	-	46.8^b^	780.0^a^		
	L	10.5^b^	3.4^a^	1.4^b^	5.4^a^	20.7^ab^	5.3^a^	7.3^a^	13.8^a^	-	42.6^ab^	759.4^a^		
4 M KOH	U	19.8^a^	3.8^a^	2.1^a^	5.9^b^	8.6^a^	5.8^a^	10.8^a^	20.1^a^	-	42.8^a^	717.8^a^		
	M	15.8^a^	2.8^a^	2.5^a^	4.0^a^	11.5^b^	5.7^a^	9.5^a^	25.8^a^	-	38.0^a^	747.5^a^		
	L	14.8^a^	3.6^a^	2.4^a^	4.8^a^	11.3^b^	5.2^a^	9.5^a^	23.6^a^	-	39.7^a^	723.0^a^		
Residue	U	41.6^a^	1.9^b^ (0.8^b^)	0.1^a^ (0.3^a^)	2.8^b^ (2.4^b^)	1.5^a^ (2.4^b^)	0.7^a^ (2.0^a^)	3.6^b^ (3.0^b^)	8.3^a^ (8.3^a^)	63.8^a^ (63.8^a^)	16.8^a^ (16.9^a^)	826.7^a^ (831.6^a^)		
	M	36.4^a^	1.5^ab^ (0.8^b^)	0.2^a^ (0.3^a^)	2.0^ab^ (2.0^ab^)	1.4^a^ (2.4^b^)	0.7^a^ (2.2^a^)	2.6^b^ (2.5^ab^)	9.1^a^ (8.9^ab^)	65.4^a^ (64.1^a^)	17.0^a^ (16.8^a^)	831.3^a^ (845.9^a^)		
	L	33.8^b^	1.0^a^ (0.5^a^)	0.1^a^ (0.1^a^)	1.3^a^ (1.5^a^)	1.5^a^ (2.1^a^)	0.9^b^ (2.3^a^)	1.8^a^ (1.6^a^)	11.8^b^ (11.6^b^)	70.2^a^ (68.9^a^)	11.6^a^ (11.4^a^)	819.7^a^ (833.8^a^)		

The yield of HEPES fraction and cell walls means the freeze dried amount of them recovered from 100 g fresh collenchyma strands

The yield of other fractions means the freeze dried amount of them recovered from 100 g freeze dried cell walls

The values in bracket of final residue and cell walls means the amount of sugars hydrolysed by H_2_SO_4_; other values are from TFA hydrolysis

“-” not detect. *DM* degree of methyl esterification of pectin (mol%), *DA* degree of acetyl esterification of cell wall (*w*/w%). *Rha* Rhamnose, *Fuc* fucose, *Ara* arabinose, *xyl* xylose, *man* mannose, *Gal* galactose, *Glc* non cellulosic glucose from TFA hydrolysis, *C-Glc* cellulose glucose, subtract TFA glucose from H_2_SO_4_, *UA* uronic acid, *TS-total monosaccharides* sum of uronic acid and neutral monosaccharides. The value averaged from duplicate or triplicate (*n* = 2 or 3)

Different letters (a, b, c) indicate significant (*P*<0.05) differences between U, M and L

The most abundant monosaccharides in the monosaccharide compositions of the HEPES fraction from the middle segment were uronic acids > Xyl > Gal > Ara. In the cell walls, the uronic acids occurred in the highest proportions, followed by cellulosic Glc (C-Glc), with Xyl and Gal in similar proportions. The monosaccharide compositions of the CDTA and Na_2_CO_3_ fractions were similar, but the Na_2_CO_3_ fractions had higher proportions of Rha, Ara and Gal, and lower proportions of uronic acids. The 1 M KOH fraction had a much lower proportion of uronic acids than the previous two fractions, but higher proportions of Xyl, followed by Glc, Man and Gal. The 4 M KOH fraction had more Glc, Gal and Fuc, but less Xyl than the 1 M fraction. C-Glc was the major component in the final residue with some uronic acids and other neutral monosaccharides.

There were some differences in the monosaccharide compositions of the cell walls and fractions from different segments. The pectin in the upper segment collenchyma walls contained higher percentage of possibly RG-I, which may be more branched due to the higher value of (Ara + Gal)/Rha (Additional file 3: Table S1). For the HEPES fraction, the upper segment had higher percentages of Xyl, Glc and Fuc, but lower percentages of Ara, Gal and Rha than the other two segments. Higher percentages of Ara and Gal were found in the 1 M KOH and 4 M KOH fractions from the upper than the other two segments. In addition, higher percentages of Xyl, Glc and Man were found in the 1 M KOH fraction from the upper segment. In the 4 M KOH fraction, the upper segment showed lower percentages of Xyl, Glc and Man than the other two segments. In the final residue, the upper segment had higher percentages of Ara, Gal and Rha, but a lower percentage of Glc than the middle and lower segments.

### Glycosyl linkage analysis

The glycosyl linkage compositions of the cell walls and fractions from the middle segments are shown in Table 2 and the deduced polysaccharide compositions in Table 3 and (Additional file 4: Table S2). These showed that the HEPES fraction contained high proportion of heteroxylans and Type II arabinogalactans, probably in the form of arabinogalactan proteins. The glycosyl linkage analysis of the cell walls was consistent with the presence of high proportion of cellulose and pectic polysaccharides, predominantly HGs (Table 3). Xyloglucan and smaller proportions of heteromannans and heteroxylans were also indicated by the linkages. The CDTA and Na_2_CO_3_ fraction were both pectin rich, as indicated by the high proportions of 4-linked Gal*p*A. The CDTA fraction contained a higher proportion of HGs than the Na_2_CO_3_ fraction, which contained a high proportion of RG-I, as indicated by the high proportions of linkages assigned to the RG-I backbone, the arabinan and (arabino) galactan side chains. The 1 M KOH fraction contained abundant HXs, XGs and smaller proportions of HM. Compared with the 1 M KOH fraction, the proportions of HXs and HMs were much lower in the 4 M KOH fraction, whereas the proportion of XGs was much higher. Furthermore, the HMs in 4 M KOH fraction had a higher proportion of Gal*p* than those of 1 M KOH fractions. The glycosyl linkage composition of the final residue was consistent with it containing mostly cellulose, but small amounts of pectin, HMs and HXs were also present.

**Table 2:** Glycosyl linkage compositions of cell walls and fractions from collenchyma strands from middle petiole segments﻿

Monosaccharides	Deduced glycosyl linkage^a^	Fractions							
		HEPES	Cell wall	CDTA	Na_2_CO_3_	1 M KOH	4 M KOH	Residue	Deduced polymer
Rhamnose	terminal*p*	1.06 (1.13)	0.09	0.31	0.69	0.27	0.21	0.04	RG-II/AG-II
	2*p*	1.68 (1.19)	1.14	2.48	5.90	2.28	1.91	0.38	RG-I backbone
	2,4*p*	0.90 (0.82)	0.36	1.04	1.96	0.83	0.71	0.06	RG-I backbone
Fucose	terminal*p*	0.29 (0.82)	0.33	0.22	0.40	1.38	3.63	-	RG-II/XGs
Arabinose	terminal*f*	6.47 (7.28)	0.94	2.14	4.16	1.85	1.21	0.35	RG-I/RG-II/AG-II/HXs
	terminal*p*	0.45 (0.42)	-	-	-	-	-	-	AG-II
	3*f*	0.40 (0.66)	-	-	-	-	-	-	RG-I
	5*f*	4.51 (4.46)	1.53	5.05	6.64	2.69	1.72	0.44	RG-I
	3,5*f*	0.91 (1.01)	0.60	1.80	3.06	1.26	0.78	-	RG-I
Xylose	terminal*p*	2.37 (3.62)	2.26	0.46	0.51	5.91	12.4	0.25	HXs/XGs/other
	2*p*	-	0.81	-	-	3.92	4.54	0.05	XGs
	4*p*	34.7 (31.9)	2.81	0.47	1.12	26.5	3.73	0.17	HXs
	2,4*p*	5.36 (5.04)	0.39	-	-	4.04	0.62	-	HXs
	2,3,4*p*	0.84 (0.66)	0.31	-	-	0.31	0.32	-	HXs
Mannose	4*p*	1.24 (0.97)	1.24	0.42	0.86	2.90	3.88	0.67	HMs
	4,6*p*	- (−)	0.78	-	-	1.33	1.86	0.06	HMs
Galactose	terminal*p*	1.69 (1.84)	0.88	0.76	1.99	3.02	4.93	0.36	RG-I/RG-II/AG-II/XGs/HMs
	2*p*	0.28 (0.94)	0.50	-	-	2.71	5.26	0.06	XGs/HMs
	3*p*	0.57 (0.81)	-	-	-	-	-	-	AG-II
	4*p*	1.14 (1.19)	1.39	1.74	3.35	1.59	1.77	0.83	RG-I
	6*p*	3.19 (2.58)	0.47	1.18	2.35	1.00	1.01	0.35	AG-II
	2,4*p*	- (0.30)	-	0.82	-	0.21	0.32	-	RG-II
	3,6*p*	5.46 (5.81)	-	-	-	-	-	-	AG-II
	3,4,6*p*	0.40 (0.55)	-	-	-	-	-	-	AG-II
Glucose	terminal*p*	1.37 (1.14)	0.43	0.29	0.73	-	0.50	0.30	Other
	4*p*	4.63 (4.45)	54.1	1.89	3.89	9.23	18.2	85.5	XGs/HMs/Cellulose/other
	2,4*p*	- (−)	0.77	-	-	-	-	0.13	Other
	3,4*p*	- (−)	0.99	-	-	-	-	0.33	Other
	4,6*p*	1.76 (3.91)	4.16	-	-	7.25	19.8	0.94	XGs/HMs/other
	3,4,6*p*	- (−)	-	-	-	-	-	0.10	Other
	2,3,4,6*p*	- (−)	-	-	-	-	0.45	0.15	Other
Galacturonic	terminal*p*	- (−)	0.12	1.30	1.21	-	-	0.05	HG/RG-II
acid	4*p*	9.21 (7.44)	19.1	70.79	54.42	12.1	8.29	6.22	HG/RG-I backbone
	2,4*p*	- (−)	-	5.04	0.85	0.13	0.10	-	RG-II
	3,4*p*	0.75 (0.91)	1.45	1.34	5.04	1.21	0.82	0.50	RG-II
	4,6*p*	0.15 (0.40)	0.39	0.44	0.86	0.30	-	-	HG
Glucuronic	terminal*p*	7.10 (6.69)	0.28	-	-	5.31	0.44	-	AG-II/HXs/Other
acid	4*p*	1.14 (1.06)	1.23	-	-	0.49	0.63	1.74	AG-II/HXs/other

Terminal Glc*p*A includes 4-O-Me Glc*p*A

Values in parentheses represent HEPES fraction from upper segment of the collenchyma in celery petioles

^a^Terminal Rha*p* deduced from 1,5-di-O-acetyl-6-deoxy-2,3,4-tri-*O*-methylrhamnitol, etc

- Not detected

**Table 3:** Deduced polysaccharide compositions of the cell walls and fractions from collenchyma strands from middle petiole segments

Polysaccharides	Composition mol %^a^							
	HEPES	Cell walls	CDTA	Na_2_CO_3_	1 M KOH	4 M KOH	Residue	Glycosyl linkages
HG	6.8 (5.8)	18	68.4	48.0	9.3	5.7	5.8	4-, 4,6-, t-Gal*p*A
RG-I backbone	5.2 (4.0)	3.0	7.0	15.7	6.2	5.2	0.9	2-, 2,4-Rha*p*; 4-Gal*p*A
Arabinan (RG-I)	6.7 (7.1)	2.7	8.7	12.8	5.2	3.3	0.4	3-, 5-, 3,5-, t-Ara*f*
(Arabino) galactans (RG-I)	1.1 (1.2)	1.4	2.0	4.3	1.6	1.8	0.8	4-, t-Gal*p*
RG-II	0.8 (1.2)	1.9	9.0	8.7	1.7	1.3	0.9	2,4-, 3,4-, t-Gal*p*A; t-Fuc*p*; t-Ara*f*; 2,4-, t-Gal*p*; t-Rha*p*
Type II Arabinogalactans (AG-II)	18.9 (20.0)	1.0	1.4	3.4	1.9	1.8	0.7	3-, 6-, 3,6-, 3,4,6-, t-Gal*p*; t-Ara*f*; t-Ara*p*; 4-, t-Glc*p*A; t-Rha*p*
HXs	47.9 (43.4)	4.2	0.5	1.1	36.9	5.5	0.2	4-, 2,4-, 2,3,4-, t-Xyl*p*; 4-, t-Glc*p*A; t-Ara*f*
XGs	4.5 (10.5)	7.5	-	-	23.1	52.0	0.8	2-, t-Xyl*p*; 4-, 4,6-Glc*p*; 2-, t-Gal*p*; t-Fuc*p*
HMs	2.5 (1.9)	4.8	0.8	1.7	10.3	15.6	1.8	4-, 4,6-Man*p*; 4-, 4,6-Glc*p*; 2-, t-Gal*p*
Cellulose	-	51.3	-	-	-	-	84.9	4-Glc*p*
Other^b^	5.7 (5.0)	4.1	2.2	4.3	3.9	7.9	2.7	4-, 2,4-, 3,4-, 4,6-, 3,4,6-, 2,3,4,6-, t-Glc*p*; t-Xyl*p*; 4-, t-Glc*p*A;

() Values represent HEPES fraction from upper segment of the collenchyma in celery petioles

Note: The deduced polysaccharides from fractions may contain only part of the glycosyl linkages listed in the last column

^a^mol% of total polysaccharides present in the fractions, calculated from the mol% of methylated alditol acetates characteristic of polysaccharides

^b^Glycosyl linkages that cannot be assigned to well-defined cell wall polysaccharides

- Not detected

### Mobilities of polysaccharides in collenchyma cell walls from different segments of the petioles

**Fig. 2:**
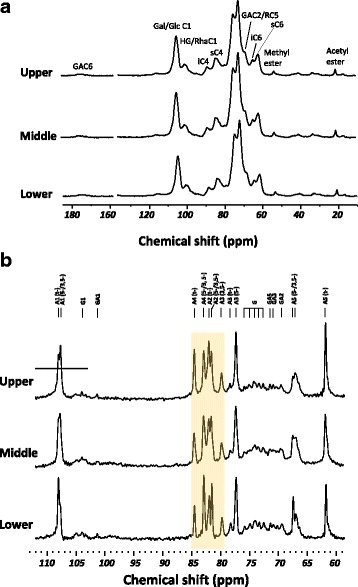
CP/MAS (**a**) and SPE/MAS (**b**) solid-state NMR of collenchyma cell walls from different petioles segments. GA-galacturonan; Gal-galactose; Glc-glucose in xyloglucan; HG-homogalacturonan; Rha-rhamnose; i-interior cellulose; s-surface cellulose. A1, arabinan C-1; G1, galactan C-1; GA1, galacturonan C-1; t-, = terminal linked; 5-, = 5-linked; 3,5-, = 3, 5-linked; 5−/3,5-, =5- and 3,5-linked

Solid-state ^13^C NMR spectroscopy can be used to differentiate cell wall polysaccharides that have different mobilities due to their molecular structures, locations and interactions with other cell wall components [36]. Both CP/MAS and SPE/MAS NMR spectroscopy were utilised to investigate the relative mobility, encompassing rigidity, of individual polysaccharides. CP/MAS spectra of collenchyma cell walls from the upper, middle and lower segments appeared very similar (Fig. 2a), indicating that the rigid and semi-rigid components (cellulose, HG, and xyloglucan) in collenchyma walls from the different segments could have similar mobility. The line width in the SPE spectra was less than in the CP/MAS spectra, suggesting the detected components have higher mobilities. The SPE spectra were dominated by arabinan signals, followed by signals from (1 → 4)-β-galactans and HG (Fig. 2b). Together with the linkage analysis (Table 3)**,** this indicates that the arabinans are particularly highly mobile, more so than the (1 → 4)-β-galactans and HG. The most evident differences among the SPE spectra form the different petiole segments are the relative peak intensities from t-Ara*f*, and (1 → 5)-Ara*f* or (1 → 3, 5)-Ara*f*, especially at C-2 and C-4.

## Discussion

In this study we found that the upper region of celery petioles elongated faster than the lower regions and was the last to stop elongating. Similar growth occurs in the hypocotyls of sunflowers (*Helianthus annuus*) and mung bean (*Vigna radiata*) [39, 40]. This suggests that collenchyma cells in the upper region of celery petioles were the last to elongate. Peripheral collenchyma cells arise from a ground meristem and pass through periclinal and anticlinal divisions before elongating [41, 42]. Previous workers showed that collenchyma cell walls thicken during cell elongation [2, 41, 43] and even post elongation [44]. However, it is not known at which stage the most wall thickening occurs. The duration of elongation and post elongation of collenchyma cells differs among the segments, which may relate to the different cell wall thicknesses but this needs further investigation. In spite of the differences in the time of the completion of elongation, wall thickness and cell size, there were only minor differences in the polysaccharide compositions of the collenchyma walls from the three segments.

The compositions of celery collenchyma cell walls are similar to those of parenchyma cell walls in most eudicotyledons, with high proportions of pectic polysaccharides in the matrix, and smaller proportions of XGs, HMs and HXs. Compared with celery parenchyma cell walls [19, 45, 46], celery collenchyma cell walls have lower proportions of pectic polysaccharides, and higher proportions of cellulose, XGs and HXs. Celery parenchyma CWs are notable for their low xyloglucan content. ﻿The higher amount of XGs in the collenchyma cell walls may be related to the mechanical function of these walls, with the XGs cross-linking the cellulose microfibrils﻿. The abundant pectic polysaccharides in the celery parenchyma walls may be involved in the maintenance of wall integrity by interacting with cellulose and other wall polysaccharides [45, 46]. Pectic polysaccharides possibly have similar roles in all primary cell walls with a high content of pectic polysaccharides.

Although the HEPES buffer-soluble fraction is only a small fraction in the present study, it has an unusual composition, with a particularly high content of heteroxylans, which are branched mostly through O-2 of Xyl*p*. The side chains possibly include Glc*p*A, 4-*O*-Me-Glc*p*A and Ara*f*, but we have no definitive evidence. The HEPES buffer-soluble fraction probably represents material in the apoplast of the collenchyma cell walls. The culture medium of cell suspension cultures can be considered as equivalent to an extended apoplast [47], and for example, the culture medium of pear (*Pyrus communis*) has also been found to contain small proportions of heteroxylans, although the main components were fucogalactoxyloglucans, a type II arabinogalactan (as part of an arabinogalactan-protein) and homogalacturonan [48]. Water soluble, highly branched heteroxylans have also been found in various plant secretions, including the seed mucilage of flax (*Linum usitatissimum*) [49] and leaf exudates of the non-commelinid monocotyledons *Phormium tenax* and *P. cookianum* [50]. Type II AGs, which also occur in the HEPES-soluble fraction, usually are part of AGPs. They have a (1 → 3)-β-D-galactan backbone substituted at O-6 by arabinosyl or galactosyl branches, with non-reducing terminal residues, including terminal L-Ara*f*, L-Ara*p*, D-Gal*p*A, D-Glc*p*A, D-Gal*p* and L-Rha*p* [51, 52]. The presence of t-Rha*p* and 4-linked Glc*p*A in the polysaccharides in the HEPES-soluble fraction, indicates α-L-Rha*p-*(1 → 4)-β-D-Glc*p*A (1→), which has been reported to occur in the AGPs of *Arabidopsis* leaves, may be present [53].

Pectic polysaccharides are the dominant matrix polysaccharides, with HG being the most abundant domain. HG can be methyl esterified, with the degree of methyl esterification being strongly correlated with the rheological properties of pectin gels. In pectins with low degrees of methyl esterification, the interaction between Ca^2+^ ions and pectic carboxyl groups (the egg-box structure) results in the formation of semi-rigid gels, which are presumed to provide mechanical support within cell walls [54], and contribute to the stability of the middle lamella [55]. The low degree of pectin methyl esterification in celery collenchyma walls may indicate that such gels are prevalent in the walls. The degree of methyl esterification of pectins is usually developmentally regulated, and shows some reduction in plant cells with low levels of wall synthesis [56]. Thus, the low degree of methyl esterification observed in celery collenchyma walls is consistent with lower levels of wall synthesis and deposition in the collenchyma cell walls in the lower segments and middle segments. Besides HG, the second most abundant pectic polysaccharide domain in celery collenchyma walls is RG-I, with (1 → 5)-α-L-arabinans as the predominant side chains, although the monosaccharide compositions of the cell walls suggested galactose is present in a comparable amount (Table 1). The functions of RG-I arabinans and galactans in plant cell walls are not known for certain; there is evidence that they can hydrogen bond to cellulose, effectively cross-linking cellulose microfibrils via the pectic polysaccharide network [57] and it has been suggested [20, 58] that they may affect the mechanical properties of cell walls by limiting the formation of egg-box structures involving Ca^2+^ cross-linked HGs. However, the proportions of arabinans and galactans are much lower in celery collenchyma cell walls than in celery parenchyma [46] cell walls, suggesting these side chains may have different roles in the walls of different cell types. Besides HG and RG-I, a RG-II domain is also present, as indicated by the presence of 3,4-linked Gal*p*A and t-Rha*p* residues [7]. However, 3,4-linked Gal*p*A could also originate from XGA [59], suggesting a XGA domain might also be present in the pectic polysaccharides of celery collenchyma cell walls. However, we have chosen not to include this domain in our list of possible polysaccharides (Table 3) because it is less widely distributed than RG-II﻿﻿.

Linkage analysis of the xyloglucan rich fractions (4 M KOH fractions) is consistent with the xyloglucans being fucogalactoxyloglucans, which occur commonly in the primary cell walls of most eudicotyledons [10]. We also found small proportions of HXs in celery collenchyma cell walls, with most being present in the 1 M KOH fractions. Early work on the HXs in the primary walls of suspension cultured cells of the eudicotyledon sycamore (*Acer pseudoplatanus*) indicated both Ara*f* and Glc*p*A (or 4-*O*-Me-Glc*p*A) were attached through O-2 of Xyl*p* in the backbone, and the HXs were glucuronoarabinoxylans [60]. However, more recently, it was found that a HX in *Arabidopsis* primary cell walls had only Glc*p*A (and 4-*O*-Me-Glc*p*A) attached directly to O-2 Xyl*p* of the backbone. Another pentose, possibly α-L-Ara*p*, was also linked through O-2 to some of the Glc*p*A residues [61, 62].

Our results also confirmed that HMs were present in small amounts in celery collenchyma cell walls. The structures of HMs from the primary cell walls of other eudicotyledons have already been elucidated, including those from blackberry (*Rubus fruticosus*) [63], kiwifruit (*Actinidia deliciosa*) [64, 65] and tobacco (*Nicotiana plumbaginfolia*) [66]. These HMs have alternating β-D-Glc*p* and β-D-Man*p* residues linked (1 → 4) as a backbone, with single α-D-Gal*p* or β-D-Gal*p*-(1 → 2)-α-D-Gal*p* residues attached mostly to Man*p*. The pattern of branching in HMs relates to their physicochemical properties, such as solubility in water, density and even interaction behaviours with other wall polysaccharides [67], which may explain why the HMs in the 1 M and 4 M KOH fractions have different degrees of branching.

The CP/MAS spectra of celery collenchyma cell walls were comparable to those obtained by Jarvis et al. [17, 18, 68]. Although the degrees of methyl and acetyl esterification of collenchyma cell walls from different segments were not identical, this could not be determined by the CP spectra. This is because most methyl and acetyl esters are highly mobile and were suppressed in CP/MAS due to inefficient polarization transfer. However, SPE/MAS spectra (Fig. 2b) suggested there is variation in the mobilities of these highly mobile arabinans in collenchyma cell walls from different segments, assuming the relaxation times are the same in each segment, indicated by the different signal patterns for terminal Ara*f*; 5- and 3, 5-linked Ara*f.* The relative amounts of terminal Ara*f*, 5- and 3, 5-linked Ara*f* in arabinans were determined by using solution-state NMR [69]. However, in solid-state NMR, both the quantity and conformation of Ara*f* can affect the intensity of signals [70], which is influenced by the degree and type of branching of arabinans. Arabinans with more branching can be expected to have relatively higher proportion of t-Ara*f* signals when detected by SPE. This is due to their increased amount as well as decreased mobility of 5−/3,5-Ara*f* (compare upper, middle and lower segments in Fig. 2b). Therefore, the arabinans in the thicker cell walls of the upper segment are probably more branched than those of middle and lower segment. As a consequence, the relative flexibility of the thicker walls may increase due to steric hindrance preventing the homogalacturonan domain coalescing [20, 58].

## Conclusions

Celery petioles elongate last in the upper region, where the collenchyma cells have smaller cross sectional areas and thicker walls compared with those from the lower regions. Cellulose and pectin are dominant polysaccharides in the collenchyma CWs, followed by XGs, HXs and HMs. The pectic polysaccharides are dominated by the HG domain, with lower proportions of RG-I with side chains mostly of (1 → 5)-α-L-arabinans rather than (1 → 4)-β-D-galactans, although more Gal was found in the monosaccharide compositions of the cell walls. Xyloglucans in the collenchyma walls are fucogalactoxyloglucans, which occur in most species of eudicotyledons. Small proportions of HXs and HMs occur in celery collenchyma cell walls, and their structures are thought to be similar to those of other HXs and HMs found in eudicotyledon primary walls. SPE/MAS combined with monosaccharide compositions indicated that the lengths and amounts of highly mobile arabinans vary among collenchyma walls from different segments of the petioles.

## Additional files

Additional file 1: Figure S1. Bright-field light micrographs (A, D, G) and VPSE micrographs (B, C, E, F, H, I) of transverse section of peripheral collenchyma strands midway along the upper, middle and lower segment of a fully expanded petiole (41 cm). Sections for bright-field light microscopy were stained with toluidine blue O (0.1%, *w*/*v*). A, B, C-upper segment; D, E, F-middle segment; G, H, I-lower segment. e, epidermis; c, collenchyma; p, parenchyma. Bars = 100 μm (A, D, G), 50 μm (B, E, H), 10 μm (C, F, I). A, D, G from the same collenchyma collenchyma strand; B, C, E were from another collenchyma strand from a different petiole; F, H, I- same collenchyma strand as B, C, E at higher magnification**.** Double headed arrows indicate the cell wall distance of thickened region between two adjacent collenchyma cells. (DOCX 1230 kb)

Additional file 2: Figure S2. Comparison of the cross-sectional area (A), cell number (B) and average cross- sectional cell area (C) and wall thickness (D) in transverse section of five different peripheral collenchyma strands midway taken along the upper (black bar), middle (red bar) and lower (blue bar) segments. Different letters (a, b, c) indicate significant difference (*P* < 0.05). P1–1, P1–2 indicate two different collenchyma strands from the same petiole. P1, P2, P3 indicate three petioles from three different plants. The wall thickness was the half distance between the thickened regions of two adjacent collenchyma cells as shown in Additional file 1: Figure S1. Thirty wall thickness were measured from two collenchyma strands from different plants, fifteen measurements for each. The averaged value and standard deviation was shown. (DOCX 40 kb)

Additional file 3: Table S1. The detail assignment of polysaccharides from celery collenchyma cell walls and fractions. (DOCX 14 kb)

Additional file 4: Table S2. Gal/Ara, (Ara + Gal)/Rha, UA/(Ara + Gal) molar ratios and (Xyl + Glc + Man) molar percentages calculated in fractions and CWs from different segments of collenchyma strands. (XLSX 39 kb)

## Abbreviations

AGP: Arabinogalactan protein
CDTA: Trans-1,2–1,2-diaminocyclohexane-N, N, N, N-tetraacetic acid
CP/MAS: Cross-polarisation with magic angle spinning
CW: Cell wall
DA: Degree of acetyl esterification
DCM: Dichloromethane
DM: Degree of methyl esterification
DTT: 1,4-Dithiothreitol
HEPES: (4-(2-hydroxyethyl)-1-piperazineethanesulfonic acid)
HG: Homogalacturonan
HM: Heteromannan
HX: Heteroxylan
MES: 2-(N-morpholino) enthanesulfonic
PMAA: Partially methylated alditol acetate
RG-I: Rhamnogalacturonan I
RG-II: Rhamnogalacturonan-II
SPE/MAS: Single pulse excitation with magic angle spinning
TFA: Trifluoroacetic acid
VPSEM: Variable pressure scanning electron microscopy
XG: Xyloglucan
XGA: Xylogalacturonan
