# Supplementary figures and images for: Polysaccharide compositions of collenchyma cell walls from celery (Apium graveolens L.) petioles
Source: BMC Plant Biol. 2017 Jun 15;17:104. doi: 10.1186/s12870-017-1046-y (PMC5472923; doi:10.1186/s12870-017-1046-y)

**Figure S1**


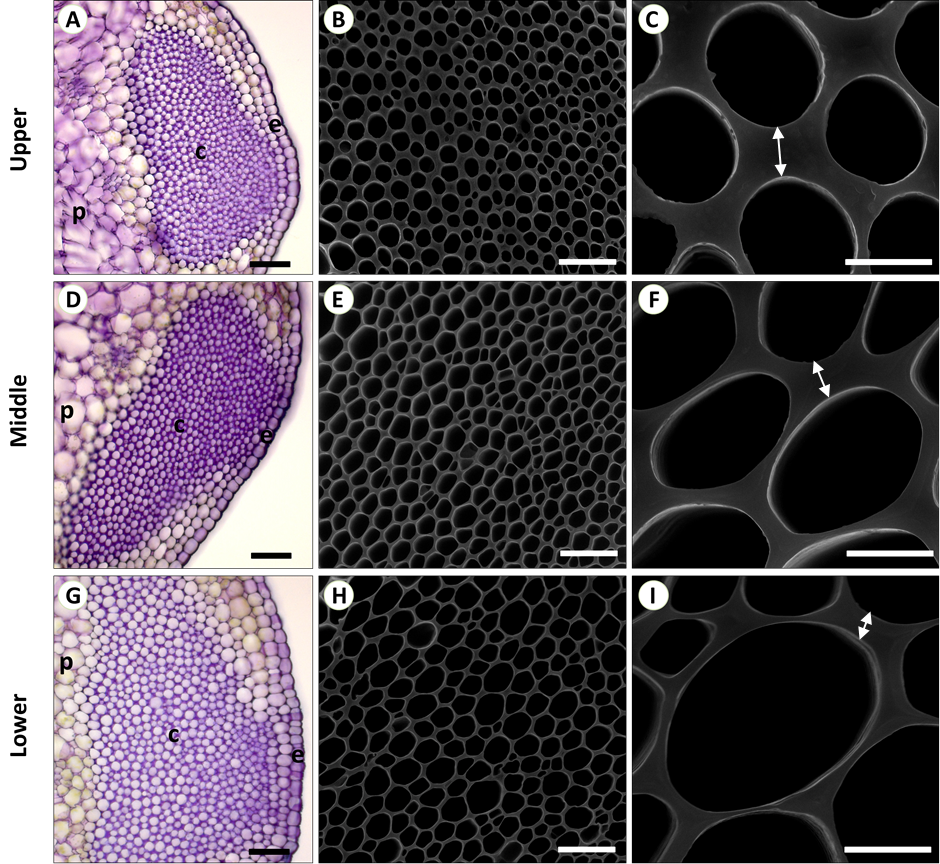

Supplement: Supplementary file 1 — Bright-field light micrographs (A, D, G) and VPSE micrographs (B, C, E, F, H, I) of transverse section of peripheral collenchyma strands midway along the upper, middle and lower segment of a fully expanded petiole (41 cm). Sections for bright-field light microscopy were stained with toluidine blue O (0.1%, w/v). A, B, C-upper segment; D, E, F-middle segment; G, H, I-lower segment. e, epidermis; c, collenchyma; p, parenchyma. Bars = 100 μm (A, D, G), 50 μm (B, E, H), 10 μm (C, F, I). A, D, G from the same collenchyma collenchyma strand; B, C, E were from another collenchyma strand from a different petiole; F, H, I- same collenchyma strand as B, C, E at higher magnification. Double headed arrows indicate the cell wall distance of thickened region between two adjacent collenchyma cells. (DOCX 1230 kb) [file 12870_2017_1046_MOESM1_ESM.docx]
