# Supplementary material for: Polysaccharide compositions of collenchyma cell walls from celery (Apium graveolens L.) petioles
Source: BMC Plant Biol. 2017 Jun 15;17:104. doi: 10.1186/s12870-017-1046-y (PMC5472923; doi:10.1186/s12870-017-1046-y)
Supplement: Supplementary file 4 — Gal/Ara, (Ara + Gal)/Rha, UA/(Ara + Gal) molar ratios and (Xyl + Glc + Man) molar percentages calculated in fractions and CWs from different segments of collenchyma strands. (XLSX 39 kb) [file 12870_2017_1046_MOESM4_ESM.docx]

**Table S2**

| **Fractions** |  |  | **Gal/Ara** | **(Ara+Gal)/Rha** | **UA/(Ara+Gal)** | **Xyl+Glc+Man** |
| --- | --- | --- | --- | --- | --- | --- |
|  | U |  | 1.4^a^ | 9.1^c^ | 1.5^a^ | 39.1^b^ |
| **HEPES** | M |  | 1.3^a^ | 7.0^a^ | 1.7^a^ | 29.6^a^ |
|  | L |  | 1.3^a^ | 7.4^b^ | 1.5^a^ | 28.4^a^ |
|  | U |  | 1.2^b^(1.2^a^) | 3.9^c^ (6.7^b^) | 4.7^a^ (4.0^a^) | 10.0^a^ (14.2^a^) |
| **CW** | M |  | 1.1^b^(1.2^a^) | 3.5^b^ (5.8^a^) | 6.4^a^ (4.7^b^) | 8.7^a^(13.5^a^) |
|  | L |  | 1.0^a^ (1.1^a^) | 3.4^a^ (5.9^a^) | 6.4^a^ (5.3^c^) | 9.3^a^ (13.2^a^) |
|  | U |  | 0.5^a^ | 4.1^b^ | 12.1^a^ | 2.5^a^ |
| **CDTA** | M |  | 0.5^a^ | 3.5^a^ | 14.1^a^ | 2.5^a^ |
|  | L |  | 0.5^a^ | 3.3^a^ | 13.8^a^ | 2.5^a^ |
|  | U |  | 0.6^a^ | 3.3^b^ | 6.7^a^ | 2.9^a^ |
| **Na_2_CO_3_** | M |  | 0.6^a^ | 2.6^a^ | 6.7^a^ | 3.4^a^ |
|  | L |  | 0.7^a^ | 2.7^a^ | 5.5^a^ | 4.3^a^ |
|  | U |  | 1.5^a^ | 4.7^b^ | 2.6^a^ | 42.8^b^ |
| **1M KOH** | M |  | 1.3^a^ | 3.9^a^ | 3.8^c^ | 36.7^a^ |
|  | L |  | 1.4^a^ | 3.7^a^ | 3.4^b^ | 39.8^a^ |
|  | U |  | 1.8^a^ | 4.4^a^ | 2.6^a^ | 34.6^a^ |
| **4M KOH** | M |  | 2.4^a^ | 4.7^a^ | 2.8^a^ | 43.0^a^ |
|  | L |  | 2.0^a^ | 4.0^a^ | 2.8^a^ | 40.1^a^ |
|  | U |  | 1.3^a^ (1.2^a^) | 3.4^a^ (6.6^b^) | 2.7^a^ (3.1^a^) | 10.4^a^ (12.8^a^) |
| **Residue** | M |  | 1.3^a^ (1.2^a^) | 3.0^a^ (5.5^a^) | 3.7^a^ (3.7^a^) | 11.2^a^ (13.5^ab^) |
|  | L |  | 1.4^a^ (1.1^a^) | 3.2^a^ (6.6^b^) | 3.9^a^ (3.7^a^) | 14.1^a^ (16.0^b^) |

The values in bracket in CWs and final residue indicates the amount of sugars from H_2_SO_4_ hydrolysis, other values are from TFA hydrolysis.

Rhamnose (Rha), arabinose (Ara), xylose (Xyl), mannose (Man), galactose (Gal); Glc, non cellulosic glucose from TFA hydrolysis;

UA, uronic acid; The value averaged from duplicate (n=2). Different letters (a, b, c) indicate significant (*P*﹤0.05) differences between U,M and L.
